# Internal variability in numerical morphodynamical experimentation

**DOI:** 10.1038/s41598-026-43401-2

**Published:** 2026-03-13

**Authors:** Lin Lin, Wenyan Zhang, Peter Arlinghaus, Hans von Storch

**Affiliations:** Institute of Coastal Systems—Analysis and Modeling, Helmholtz-Zentrum Hereon, 21502 Geesthacht, Germany

**Keywords:** Geomorphology, Climate sciences, Ocean sciences

## Abstract

It has long been recognized that the morphodynamics of coastal bays are not fully deterministic but instead exhibit inherent uncertainty. This intrinsic variability is reminiscent of internal variability in climate systems, arising not only from dynamical instabilities but also from the integration of random disturbances. Traditionally, uncertainty in morphodynamic behavior has been investigated through stability analyses. However, traditional assessments of stability have largely focused on the stability property of low-dimensional analysis. In realistic dynamical numerical models, morphodynamic systems are rarely low-dimensional. In these cases, it is often more appropriate to regard the uncertainty as a manifestation of stochasticity. For real-world systems, this raises an important question: when does an observed development lie outside the range of intrinsic variability, implying the influence of an external driver? Answering this is considerably more complex than in controlled numerical experiments, where a single parameter or process can be isolated and modified. In this paper, we examine uncertainty in a relatively simple morphodynamic numerical model of a coastal bay. We show that minor changes in the initial conditions—such as the tidal phase at model initialization—can lead to differences among ensemble members, substantial in local features (e.g., the fine-scale structure of channels) and less so in overall properties, such as mean bay depth and channel number count. Consequently, a robust evaluation of numerical experiments, such as those testing the effects of altered parameterizations, requires explicit estimates of the system’s inherent uncertainty so that the “signal” generated by experimental changes can be distinguished from internal variability (“noise”).

## Introduction

Understanding and predicting the evolution of morphodynamic systems has long been recognized as a fundamental challenge. These systems frequently display limited predictability, as their observed trajectories cannot always be uniquely explained; alternative developments often appear equally plausible. The stability analysis is a key tool for predicting the equilibrium configuration of tidal inlets, ebb-tidal deltas, channels and flats^1^. Early work—most notably summarized in the classical review^2^— highlighted that such variability largely arises from intrinsic instabilities embedded in simplified, low-dimensional morphodynamic formulations that involve only a small number of governing variables.

Recent theoretical advances have expanded this perspective by reproducing idealized fractal branching networks and revealing the so-called *laziness phenomenon*, in which increased channelization paradoxically diminishes the spatial efficiency of the network^3^. These free-patterning behaviors arise spontaneously from instabilities at the liquid–solid interface, appearing as interfacial waves that modulate either bed elevation or channel alignment^4^. Linear stability analyses commonly yield algebraic dispersion relations, where bar-forming mechanisms are driven by frictional influences and counteracted by the lateral component of gravity, while nonlinear interactions introduce additional complexity to the ensuing morphodynamic evolution.

With the advent of more “quasi-realistic” numerical models^5^, morphodynamic research has moved beyond conceptual formulations toward systems featuring multiple state variables and spatially resolved domains—effectively introducing many additional degrees of freedom. While conceptual morphodynamic models have been successful in describing the spontaneous emergence of patterns through deterministic instabilities, a key challenge remains: determining whether small-scale features arise from internal variability or from externally imposed forcing. Early sensitivity studies investigated how coastal morphodynamic systems respond to variations in fluvial and marine forcings—considering factors such as bottom slope, tidal current strength, sediment characteristics^6^, sea-level rise^7^, coastal vegetation^8^, as well as the influence of initial planform geometry^9^ and initial bathymetric conditions^10^. Although these studies documented how morphodynamic systems respond to variations in external forcings, they did not explicitly frame their results in terms of the dual influence of intrinsic system instabilities and external conditions. In numerical modeling, this distinction emerges as two forms of variability: (1) system responses to external forcing (the “signal”), and (2) internally generated variability that cannot be traced to external drivers. Yet, the systematic use of ensemble simulations and statistical techniques to separate internally generated fluctuations from externally forced responses has not been explored in morphodynamic modeling. Please note that the ensemble simulations in this study share identical model configurations, except for the different initialization times. They are distinct from ensemble approaches that perturb model parameters to account for parametric uncertainty^11^, or from ensembles used to assess the rapid loss of skill in short-term forecasts^12^. In these latter two types of ensemble simulations, the issue of separating signal from noise—which is the focus of this paper—is rarely addressed.

When internal variability is present in a dynamic system, numerical experiments designed to assess the effects of adding or modifying processes (e.g., parameterizations) face the well-known challenge of *detection*^13,14^. The core issue is to determine whether observed changes in simulated behavior—such as coastal erosion rates, channel migration pathways, or sediment deposition patterns—can be attributed solely to the system’s inherent internal variability, or whether they reflect a statistically significant response to external perturbations introduced through the model configuration. This discrimination typically relies on formal statistical hypothesis testing, which requires an explicit estimation of the magnitude and structure of internal variability.

In numerical experiments, this task is relatively straightforward because the factor responsible for potential deviations from a baseline state is known by construction: it is the imposed experimental change. The situation is fundamentally different when evaluating changes in the real world, where a multitude of external drivers may be plausible. In such cases, a procedure is required to identify—from many conceivable causes—the most likely contributors to the observed change, a process referred to as *attribution*^14,15^. In both contexts—numerical experimentation and interpretation of observed environmental change—knowledge of the underlying level of unforced internal variability is essential for distinguishing signal from noise. For numerical models, this variability can be estimated by constructing ensembles of undisturbed simulations (for example, by imposing small perturbations to initial conditions). For observations, however, quantifying the background variability is considerably more challenging, making detection and attribution correspondingly more complex.

The concept of *detection and attribution* was originally developed within climate science to formally identify and quantify the drivers of observed environmental changes^15–17^(subject of the 2021 Nobel Prize in Physics). Early applications focused primarily on distinguishing anthropogenic signals in global and regional temperature records from natural internal variability^18^, laying the methodological foundation for subsequent attribution studies across the coupled climate system. Over the following decades, detection and attribution methods expanded to encompass a wide range of atmospheric and oceanic processes, including sea-surface temperature trends^19^, sea-level rise^20^, ocean heat uptake^21^, and large-scale circulation changes^22^. By integrating ensemble simulations, statistical fingerprinting, and explicit uncertainty quantification, the detection–attribution framework has become a cornerstone for separating externally forced responses from internal variability in the climate system.

In contrast, previous morphodynamic numerical experiments have predominantly relied on pairs of simulations: a “control” simulation representing unchanged conditions and an “experimental” run in which one or more parameters were modified. The difference between these two simulations was then interpreted as the effect of the imposed change. However, this approach neglects the uncertainty associated with internal variability and ignores the possibility that the simulated differences may simply reflect unforced fluctuations. Such pairwise comparisons are therefore insufficient—an issue recognized since the mid-1970s in atmospheric science and later reinforced in oceanography, where the need to account for internal variability has long been established.

The intensities of internal variability typically escalate with increased degrees of freedom. For instance, internal variability amplifies when the spatial model resolution is augmented, as observed when comparing eddy-resolving simulations with a non-eddy-resolving simulations^23^. Consequently, it is more compelling to employ a simplified model to substantiate the presence of internal variability. As external forcings exhibiting clear interannual variations have been eliminated, we adopt the latter approach, specifically by introducing a minor perturbation at the initiation of each ensemble simulation (see the Methods section).

In this study, we limit our focus to a relatively simple model, subject to only tidal forcing while excluding other external forcings, including wind forcing and heat flux. We describe the internal variability is present even within such a simplified model framework. To do so, we construct ensembles of simulations which are identical in set-up, but deviate by minuscule initial differences, namely the phase of the tide in the initial state.

Consequently, it is more compelling to employ a simplified model to substantiate the presence of internal variability. As external forcings exhibiting clear interannual variations have been eliminated, we adopt the latter approach, specifically by introducing a minor perturbation at the initiation of each ensemble simulation (see the Methods section).

We first assess whether internal variability influences the morphodynamic evolution. To this end, we employ ensemble numerical simulations, an established approach for quantifying internal variability. A small perturbation is introduced at the beginning of each experiment by shifting the tidal phase at start time. Our working hypothesis is that these short-term disturbances may amplify over multi-decadal integration, leading to divergent morphodynamic states. Two outcomes are possible: the perturbations may dissipate after some time, yielding similar long-term behavior across ensemble members, or they may grow and produce substantially different trajectories. The spread among ensemble members therefore reflects the range of possible evolutions under identical external forcing and thus provides a measure of internal variability.

Details of the morphodynamic model configuration and the ensemble design are provided in the section Method configuration and ensemble simulation design. The Results section presents the divergent trajectories of the four ensemble members. Finally, the Discussion and conclusion section synthesizes the principal findings, evaluates limitations, and considers whether the system’s behavior is better characterized by a chaotic paradigm or by a stochastic climate model framework.

## Model configuration and ensemble simulation design

### Model setup

**Fig. 1:**
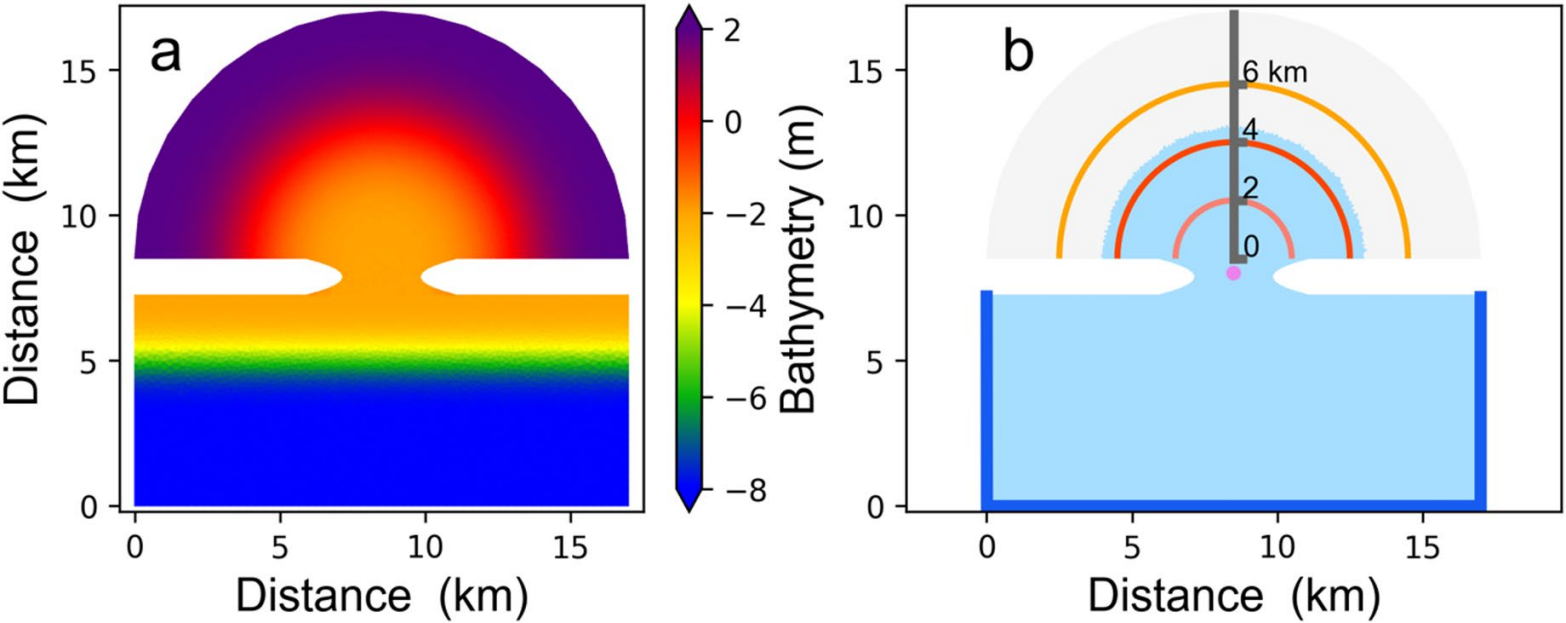
The bathymetry of the model domain (**a**) and semicycles to calculate the average depth of the channel and the number (**b**). In Fig. 1b, light blue and grey areas indicate regions below and above zero water depth, representing sea and land, respectively. A pink dot marks the centre of the tidal inlet. A dark grey line shows the section for the Hovmöller diagram (Fig. 5), with distance scales from the tidal inlet. Around the pink dot, circles with radii of 2 km (light pink), 4 km (red), and 6 km (orange) are shown. Semicircles from 0 to 8 km at 0.1 km intervals are used for metrics, with three semicycles illustrated for demonstration.

**Fig. 2:**
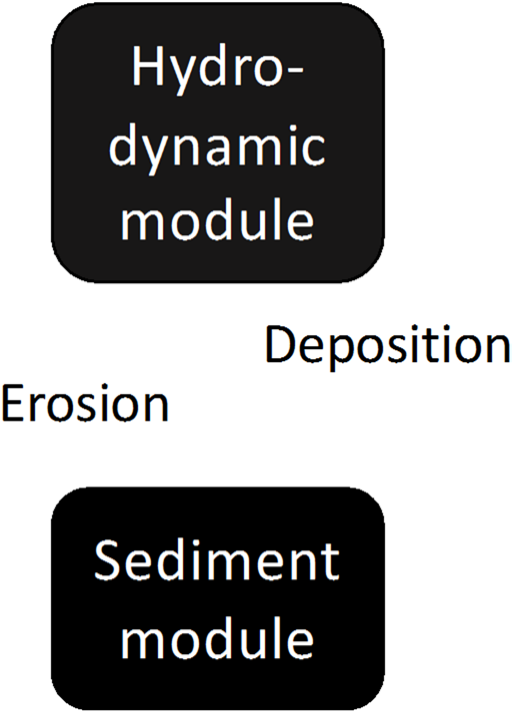
Schematic overview of the components of the numerical model and their coupling. The hydrodynamic and sediment modules are from the SCHISM model.

To have a general understanding of the internal variability in morphology development, we have adopted an idealised coastal morphology which incorporates an offshore area, a tidal inlet, and a tidal basin to mimic typical tidal embayments (Fig. 1). This model setup has been used in previous research^24^. We use the Semi-implicit Cross-scale Hydroscience Integrated System Model (SCHISM) to simulate the hydrodynamics and morphodynamics^25^. The SED3D sediment model is part of SCHISM^26^. A schematic overview of the components of the numerical model and their coupling is shown in Fig. 2. The hydrodynamic module in SCHISM drives sediment transport by providing flow properties such as velocity and shear stress, which control erosion and deposition. The sediment module simulates sediment settling, resuspension, and transport, feeding back changes in bathymetry and hydrodynamic conditions.

This dynamic coupling ensures realistic interactions between the flow and sediment processes. The k-kl closure scheme is implemented in the study^27^.The model domain covers an area of 17 by 17 ${km}^{2}$ illustrated in Fig. 1. The lines marked in dark blue (Fig. 1b) in the model domain is the seaward boundary, and the other boundaries are solid boundaries. The upper semicycle region is the basin region, and the lower part in the model domain is the ocean, a tidal inlet connects the bay to the open ocean, allowing the exchange of tidal waters between these two bodies. Within the tidal inlet and basin region, the horizontal resolution of grid cells is approximately 150 m, gradually reducing to 300 m at the seaward boundary. Even if SCHISM is a 3-dimensional model, in our study, we use a 2-dimensional model setup. An overview of the essential model parameters is provided in Table 1.

The initial seabed material consists of uniformly distributed sands with ${D}_{50}=120\mu m$. with a settling velocity, critical shear for erosion and erosion rate of 0.01 $mm{s}^{-1}$, 0.14 $Pa$, $1\times{10}^{-3}s{m}^{-1}$.

Along the seaward boundary, the model is forced by a semidiurnal tide with an amplitude of about 1.5 m, identical for all points along the boundary. The amplitude of the tidal forcing is chosen to be similar to the average tidal amplitude of 1.42 m at the mouth of a tidal Jade Bay in the German Wadden Sea measured by the *Bundesanstalt für Gewässerkunde (BFG)*. To reduce the complexity of the model, Coriolis force, wind-waves, effect of temperature and seasonality are excluded, even if it is clear that in particular the Coriolis force influences the velocity pattern massively^28^, but for our purpose, namely the demonstration of emerging spatially larger and long-term variations by minuscule initial disturbances, this is not necessary.

To save computer time and to have a stable state of coastal morphology, we use morphological acceleration to update the change in bed level. Sensitivity runs regarding the value selection of a numerical acceleration of 40, as described by Arlinghaus et al.^24^.

**Table 1:** Configuration of model parameters.

Parameter	Configuration
Domain size	17$\times$17 ${km}^{2}$
Resolution	150–300 $m$
Grid type	Triangles
Vertical layers	1
Bed layer	1
Bed layer height	12
Time step	120 s
Morphological acceleration factor	40

### Ensemble simulation designs

**Fig. 3:**
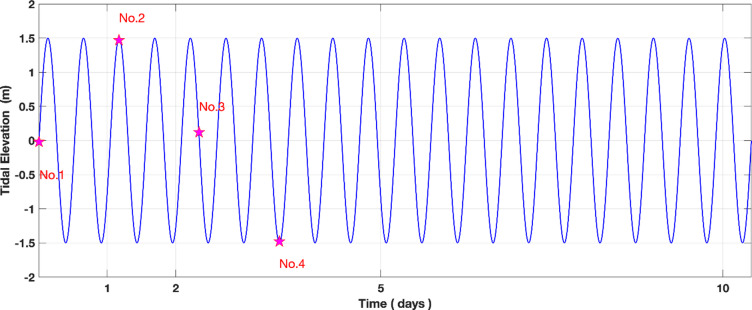
Tidal forcing along the open seaward boundary. Nos. 1, 2, 3, and 4 are the numerical simulation members in the ensemble. The pink stars mark the start time of the model simulation members.

Four numerical simulations were performed, each running for 240 years, with slight variations in initial conditions achieved by introducing small temporal shifts (a few days) at the start time of each simulation. The simulations were initiated on the first, second, third, and fourth days of the first year, respectively (see Fig. 3). The model output after 100 years was used for subsequent analysis.

It is crucial to acknowledge that the degree of variability generated by various initial conditions is contingent upon the system’s ability to allow perturbations to expand. When an anomaly or disturbance is introduced, it may quickly fade away or continue to evolve. This study aims to explore if the morphological system enables a minor perturbation to progress, and if so, to analyze the amount of divergence over time and assess whether these deviations stabilize after extended development.

### Channel detection and extraction

In the discipline of morphology, a channel is characterised as a natural or artificial depression or conduit through which water flows. These channels are commonly V- or U-shaped, resulting from hydrodynamic forces and sediment transport. Our numerical modelling results exhibit complex networks of channels, and to accurately assess morphological changes, we implement a channel detection methodology based on image analysis using OpenCV (Open Source Computer Vision Library) and adaptive thresholding^24,29^. This approach enables the digitisation of both the number and depth of channels, which are then used for comprehensive statistical analysis.

## Results

**Fig. 4:**
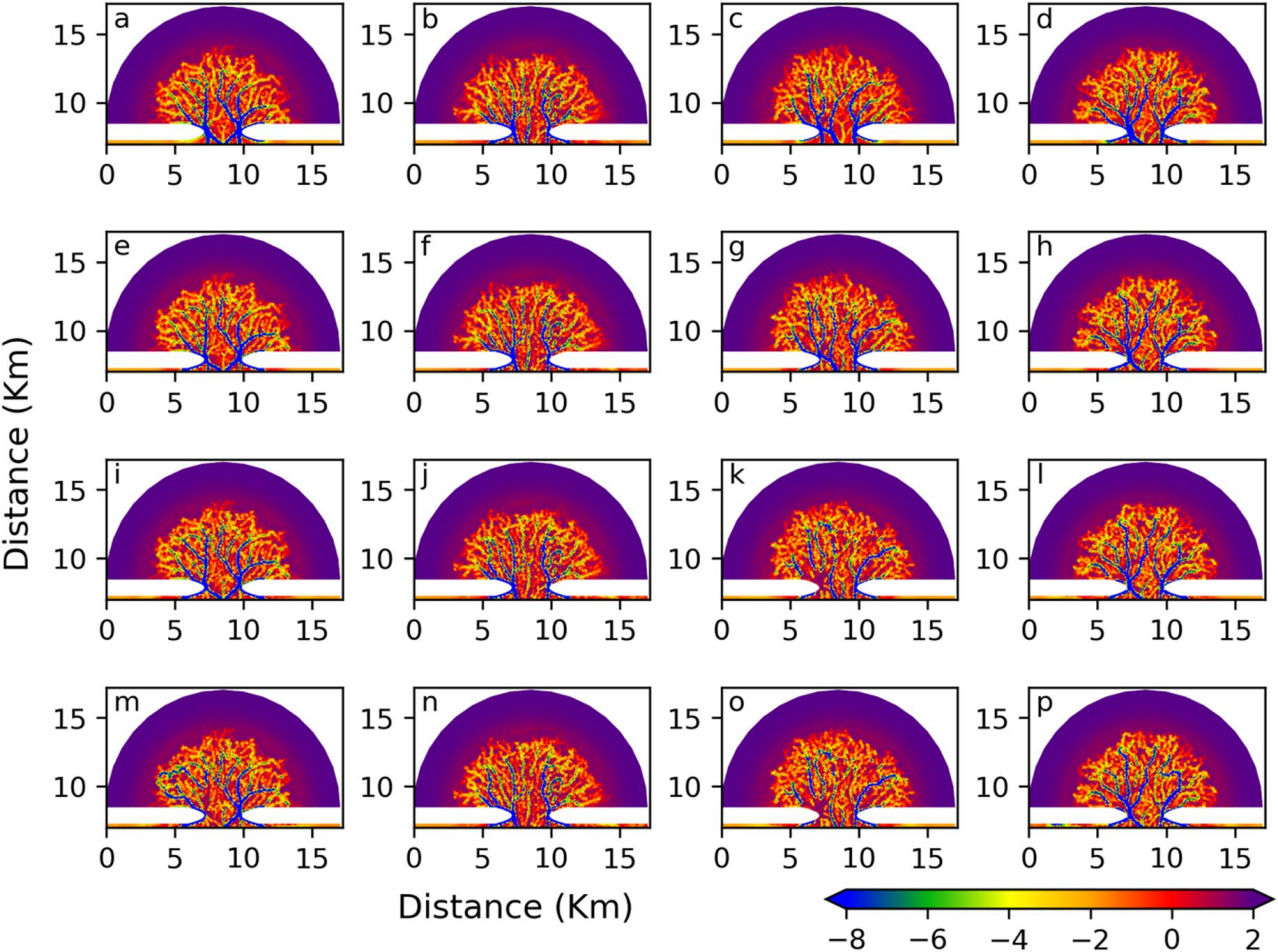
The simulated evolution of the morphology (depth of the seabed). Figures **a**–**d**: 120 year; Figuures **e**–**h**: 160 year; Figures **i**–**l**: 200 year; Figures **m**–**p**: 240 years. The first, second, third, and fourth columns represent columns Nos. 1, 2, 3, and 4, respectively. (Unit: m).

To illustrate the presence of internal variability within the ensemble, the simulated morphology for each ensemble member is displayed at 120, 160, 200, and 240 years (Fig. 4), showing how the members diverge over time. Over several decades, in the open ocean region, the depth of the seabed increases in areas where channels develop, while it becomes shallower in regions where no channels form. This variation in morphological development is particularly pronounced near the tidal inlet. The observed changes in bedform, erosion in channelised areas and deposition in adjacent non-channelised areas, reflect the redistribution of sediment between the seabed and seawater. Sediment is transported to areas surrounding the channels, leading to deeper depth in the seabed.

In the tidal basin, multiple channels develop, evolving in terms of depth, length, and number. Figure 4 shows that the depth and number of channels grow over time and significant differences emerge between ensemble members, particularly with respect to the number, depth, and bifurcation patterns of the main channels. These variations demonstrate that small initial perturbations can evolve the sea bed from the initial radially uniform states into distinct morphological states as the model integrates over time. In the following section, we analyse the statistical characteristics of these results, which represent the response of the signal.

**Fig. 5:**
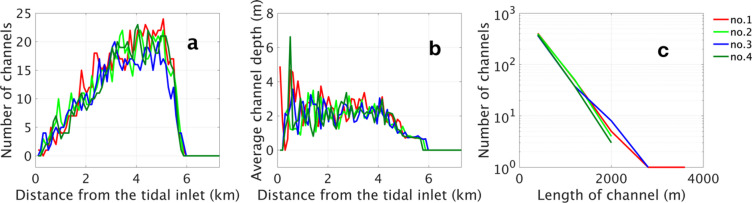
In year 240, the variation in the number of channels with distance from the tidal inlet (**a**), the variation in average channel depth with distance from the tidal inlet (**b**) and the relationship between average channel depth and channel length (**c**) between all numerical simulation members.

We further investigate the variation in the number and depth of channels with distance from the tidal inlet (Fig. 5a,b) and assess the relationship between channel number and length in year 240 to quantify deviations among ensemble members (Fig. 5c).

The number of channels and the average channel depth are calculated along semicircles of varying radii. In Fig. 5a,b, the number and average length at each semicircle are plotted for the final year of 240. In both ensembles of curves, marked in different colours for the different simulations, the general form is similar, with few channels near the entrance maximum at about 5 km and no channels after 6 km, where the bay is either very shallow or even dry. There are notable variations, which may be mostly due to the discrete character of the channels and not as an indication of temporal variations. A similar result is shown in Fig. 5b: In most simulations, the depth of the channel increases from 0 to 1 km from the tidal input, stabilising at around 2 m between 1 km and 4 km, and then gradually decreases with further distance. Beyond 6 km, the channel depths approach zero, as this area is near the coast, where seawater hardly reaches, preventing channel formation.

Figure 5c demonstrates the correlation between channel number and length, indicating a higher prevalence of shorter channels compared to longer ones. The number of channels corresponding to a specific length remains generally uniform across ensemble members, with the exception of some members that include channels extending beyond 2000 m. Although Fig. 5 refers to year 240, analogous trends are discernible in other simulation years. These observations imply that, despite minor morphological variations, the members of the ensemble predominantly display consistent statistical features.

**Fig. 6:**
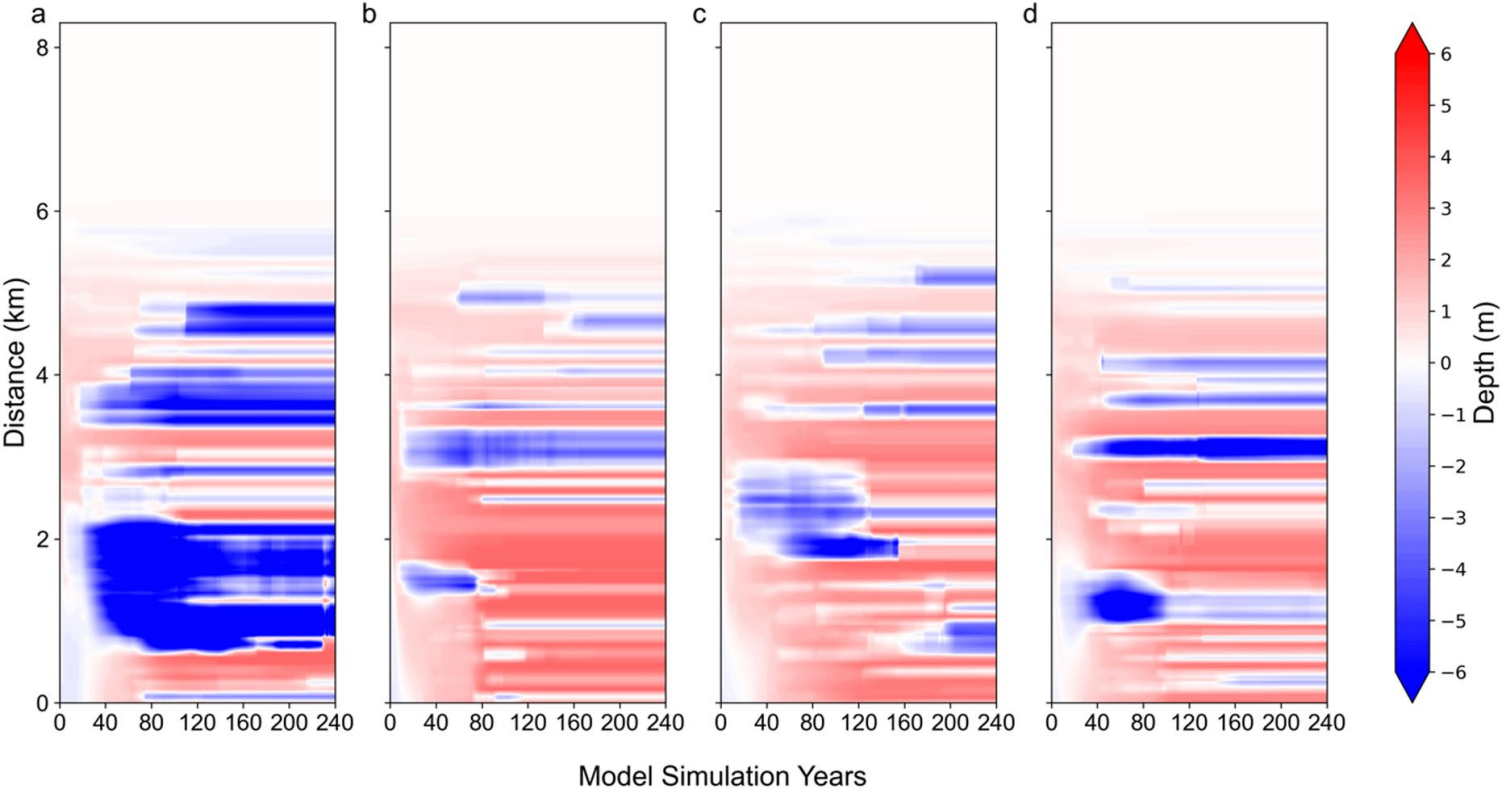
The Hovmöller diagram of the change in seabed depth along the section (as shown as a dark grey vertical line in Fig. 1b) in the four members of the ensemble.

To demonstrate the continuous large-scale morphological evolution over time along a specific section (denoted by the dark grey vertical line in Fig. 1b), a Hovmöller diagram is presented in Fig. 6. The change in seabed depth is depicted in Fig. 6, as opposed to the actual seabed depth illustrated in Fig. 4, to more clearly reveal erosion and deposition. Initially, seabed changes are relatively minor (approximately zero) during the first 20 years following the start of the model. However, significant variations in seabed depth emerge subsequently. Along the y-axis, erosion (blue area in Fig. 6) and deposition (red) occur at various locations within the ensemble members. Specifically, in the member of the ensemble No.1 (Fig. 6a), a channel is located at the centre of the basin (the region surrounding the dark grey vertical line), resulting in the formation of a pronounced erosion zone at the centre of the basin. In contrast, other ensemble members also exhibit channel development; however, the primary channel does not coincide with the dark grey vertical line that delineates the basin’s centre. Within each member of the ensemble, changes in seabed depth occur in the region that extends from 0.5 to 6 km from the tidal inlet. In contrast, in the area located 6 to 8 km from the tidal inlet, alterations in the seabed remain minimal throughout the 240-year simulation. This observation aligns with the absence of channel formation proximate to the coastal solid boundary, as depicted in Fig. 4. Furthermore, the large-scale discrepancies among ensemble members persist during the simulation, thereby indicating that the system provides conditions conducive to the amplification and maintenance of disturbances.

A remarkable and intriguing detail is that Fig. 6 is mostly made of horizontal stripes, i.e., in most cases if after 20 or 40 years a location has been subject to erosion, this erosion is almost never balanced out. If a location is marked in blue after 40 years, it remains mostly so in the year 240. The same applies to reddish-marked locations. Such changes from accretion to erosion would establish a kind of low-frequency variability. Thus, very little variability appears locally. The channels do not change position once they have formed. We come back to this in the final Sect. 4.

## Discussion and conclusion

This study investigates the influence of minimal initial perturbations on a simple two-dimensional morphodynamic model subjected to mono-frequency tidal forcing over a period of 240 years. Early disturbances are introduced by slightly shifting the temporal start of the model, with no additional perturbations applied thereafter. These initial disturbances are not arbitrarily imposed but arise naturally from insignificant differences in starting time. Ensemble simulations reveal that channel formation occurs consistently across all runs; however, significant variability is observed in the precise locations and finer-scale characteristics of the channels among ensemble members. Notably, once a branch-shaped channel develops in a given area within any ensemble member, its large-scale spatial configuration remains largely stable over time.

In geomorphology, small initial changes were found to sometimes produce disproportionately large and long-lasting effects^30^. In numerical models, small-amplitude perturbations on the seabed were introduced, for example, by adding a random value to the depth of each grid cell and lead to changing pattern formation¹. Many previous numerical studies^31–35^ have claimed to explore the importance of such variations in bedform evolution. For instance, friction is known to play a key role in amplifying bottom perturbations^7^, while sensitivity to initial bathymetry^7,36^ and differences in initial topography influence the trajectories of simulated systems in phase space^36^. However, these studies disregarded the presence of random variations due to internal dynamics.

The ensemble strategy used in this study—slightly shifting the initial state in time—differs from previous work, which often generated ensemble members by deliberately modifying the model configuration. In practical numerical simulations, choosing an initial state a few days earlier or later is almost unavoidable. Consequently, the modeled trajectory of a morphodynamic system may not be perfectly reproducible when a different initialization phase is used.

Morphodynamic systems exhibit pronounced intrinsic variability—self-organized fluctuations that can arise even under constant external forcing—posing fundamental challenges for interpreting numerical experiments and sensitivity analyses. Implementing ‘detection’ methodologies in morphodynamic modeling provides a rigorous framework to distinguish externally forced morphological changes, such as those driven by sediment properties^6^, sea level rise^7^, or coastal vegetation^8^. Integrating detection principles into morphodynamic studies enhances model interpretability and strengthens the robustness of sensitivity experiments. In this paper, we emphasize the importance of internal variability and the necessity of distinguishing the effects of external forcing from those of internal variability. We do not present a detection-and-attribution case study here; such analysis will be addressed in a forthcoming paper.

Furthermore, numerical model results indicate that once distinct morphological trajectories emerge within the first 20–40 years, the large-scale characteristics of channels tend to persist throughout the simulation period. For instance, a channel forming near the center of a bay often retains its position over time. In this context, the initial development of channels from a radially uniform bedform resembles a random walk; however, once primary channels are established, subsequent trajectories remain constrained near the state defined by these main channels.

Notably, these dynamics resemble those of the Lorenz system^37^, where tiny perturbations can lead to substantially divergent system states. This contrasts with stochastic climate models, which typically incorporate white-noise forcing into red-noise spectra of large-scale systems. While white noise exhibits no temporal correlation, red noise reflects persistence: anomalies in the system tend to endure over time rather than fluctuate randomly.

However, we suggest that once the major channels have formed, the system is becoming insensitive to small disturbances – a stable state is reached in the word of Phillips^30^. It needs to be seen if stability of minor channels emerges at a later time. Thus, the variability in the formation of diverse, yet statistically equivalent channels may depend on the presupposition of a uniform initial seabed, as illustrated in Fig. 1a. It is likely that if the same numerical experiment was conducted with primary channels already present in the initial condition, the variability across ensemble simulations might diminish significantly. A potential limitation of our research could be that the observed behaviour is contingent on the quality of the initial state, specifically whether significant channels are already present or, as in our setup, whether the initial seabed is smooth.

While the present ensemble simulations provide robust evidence for the role of internal variability, it is important to acknowledge the limitations of the modeling framework. The morphodynamic model employed here should not be interpreted as a direct representation of natural systems. Current morphodynamic models remain constrained by several technical bottlenecks that limit their ability to capture realistic landscape evolution. For example, the present setup does not account for dynamic channel meandering, avulsion, or fragmentation. If such processes were active, the system would likely exhibit far less stability than described here, where a dominant channel forms and persists over time. In reality, channels migrate and reorganize continuously, making the persistence of a single pathway highly uncertain.

Therefore, although the simulated system evolves toward a state that can be described as a dynamic equilibrium, this state should be viewed as an emergent property of the model configuration rather than a faithful depiction of natural morphodynamic equilibrium. Regarding ensemble size, previous ocean ensemble simulation studies have employed larger ensembles^38,39^. For our purposes—demonstrating internal variability in regional models and highlighting the importance of ensemble simulations—a larger ensemble is not necessary.

Despite these simplifications, the model offers valuable conceptual insights into how intrinsic variability can influence the co-evolution of flow and morphology.

## Data Availability

The source code of the used SCHISM is available at https://github.com/schism-dev/schism. The Matlab and Python code used in the paper is available at [10.5281/zenodo.15072252](https:/doi.org/10.5281/zenodo.15072252).
